# Supplementary material for: Precocious expression of Blimp1 in B cells causes autoimmune disease with increased self‐reactive plasma cells
Source: EMBO J. 2018 Nov 29;38(2):e100010. doi: 10.15252/embj.2018100010 (PMC6331720; doi:10.15252/embj.2018100010)
Supplement: Supplementary file 1 — Appendix [file EMBJ-38-e100010-s001.pdf]

Appendix

Table of contents

1. Appendix Supplementary Methods
2. Appendix Figures
3. Appendix Supplementary References

1. Appendix Supplementary Methods

Mice

The following mice were maintained on the C57BL/6 genetic background: *Prdm1*$^{ihCd2/ihCd2}$ (Minnich et al., 2016), *Prdm1*$^{Gfp/+}$ (Kallies et al., 2004), Eβ$^{-/-}$ (Bouvier et al., 1996), J_HT (Gu et al., 1993), *Rosa26*$^{BirA/BirA}$ (Driegen et al., 2005), *Pax5*$^{iGfp/iGfp}$ (Fuxa and Busslinger, 2007), *Rag2*$^{-/-}$ (Shinkai et al., 1992), *Fasl*$^{gld/gld}$ (Takahashi et al., 1994) and transgenic *Vav-Bcl2* (Ogilvy et al., 1999) mice. All animal experiments were carried out according to valid project licenses, which were approved and regularly controlled by the Austrian Veterinary Authorities.

Generation of *Prdm1*$^{\Delta 3'U(90)/\Delta 3'U(90)}$ mice

The 3'UTR in the endogenous *Prdm1* locus was deleted by CRISPR/Cas9-mediated genome editing (Yang et al., 2013). For this, *Cas9* mRNA was co-injected with two specific sgRNAs (linked to the scaffold tracrRNA) into mouse zygotes (C57BL/6 x CBA), as previously described (Yang et al., 2013). The sgRNAs and PCR genotyping primers are shown in Table EV3. The PCR reaction amplified a 399-bp and 218-bp fragment for the wild-type and the 3'UTR-deleted allele, respectively. The *Prdm1*$^{\Delta 3'U(90)/+}$ allele was backcrossed at least 5 times to the C57BL/6 genetic background.

Antibodies

The following monoclonal antibodies were used for flow cytometry: B220/CD45R (RA3-6B2), CD3ε (145-2C11), CD4 (GK1.5), CD5 (53-7.3), CD8α (53-6.7), CD11b/Mac1 (M1/70), CD19 (1D3), CD21 (7G6), CD22 (Cy34.1), CD23 (B3B4), CD25 (PC61), CD28 (37.51), CD40 (3/23), CD44 (IM7), CD49b (DX5), CD62L (MEL-14), CD69 (H1.2F3), CD80 (16-10A1), CD86 (GL1), CD90.2/Thy1.2 (30-H12), CD95/Fas (Jo2), CD117/c-Kit (2B8), CD127/IL7Rα (A7R34), CD135/Flt3 (A2F10), CD138 (281-2), CD279/PD-1 (J43), CXCR5 (2G8), F4/80 (CI:A3-1), GL7 (GL7), Gr1 (RB6-8C5), IgD (11-26c.2a), IgM (II/41), Ly6C (6C3), Ly6D (49-H4), MHCII (M5-114), NK1.1 (PK136), Sca-1 (D7), TCRβ (H57-597), TCRγδ (GL3) and human CD2 (RPA-2.10).

For detection of intracellular proteins by flow cytometry using the Foxp3 staining buffer set (eBioscience; 00-5523-00), the following antibodies were used: Bcl6 (K112-91), Blimp1 (5E7) and cleaved Caspase 3 (5A1E). For immunoblot analysis of nuclear extracts, the following antibodies were used: rat monoclonal antibody (mAb) to Blimp1 (5E7) and mouse mAb to Tbp (3TF1-3G3; Active Motif). The following antibodies were used to detect histone modifications by

ChIP-qPCR: H3K4me1 (rabbit polyclonal Ab; Abcam; ab8895) and H3K27ac (rabbit polyclonal Ab; Abcam; ab4729).

Definition of cell types by flow cytometry

Lymphocyte populations were gated as follows: LMPPs (Lin$^-$Flt3$^+$IL7Rα$^-$B220$^-$c-Kit$^+$Sca-1$^+$), ALPs (Lin$^-$Flt3$^+$IL7Rα$^+$B220$^-$c-Kit$^+$Ly6D$^-$), BLPs (Lin$^-$Flt3$^+$IL7Rα$^+$B220$^-$c-Kit$^+$Ly6D$^+$), total B cells (CD19$^+$B220$^+$), pro-B cells (CD19$^+$B220$^+$IgM$^-$IgD$^-$c-Kit$^+$CD25$^-$), pre-B cells (CD19$^+$B220$^+$IgM$^-$IgD$^-$c-Kit$^-$CD25$^+$), immature B cells (CD19$^+$B220$^+$IgM$^+$IgD$^-$), recirculating B cells (CD19$^+$B220$^+$IgM^{lo}IgD$^+$), follicular B cells (CD19$^+$B220$^+$CD21$^{int/lo}$CD23^{hi}), marginal zone B cells (CD19$^+$B220$^+$CD21^{hi}CD23^{lo}), germinal center B cells (CD19$^+$B220$^+$GL7$^+$Fas$^+$), peritoneal B-1a cells (CD19$^+$CD23$^-$CD5$^+$), peritoneal B-1b cells (CD19$^+$CD23$^-$CD5$^-$), peritoneal B-2 cells (CD19$^+$CD23$^+$CD5$^-$), plasmablasts (CD138$^+$CD22^{lo}), plasma cells (Lin$^-$B220$^{int/–}$ CD138$^+$CD28$^+$), DN1 thymocytes (CD4$^-$CD8$^-$TCRβ$^-$CD44$^+$CD25$^-$), DN2 thymocytes (CD4$^-$CD8$^-$TCRβ$^-$CD44$^+$CD25$^+$), DN3 thymocytes (CD4$^-$CD8$^-$TCRβ$^-$CD44$^-$CD25$^+$), DN4 thymocytes (CD4$^-$CD8$^-$TCRβ$^-$CD44$^-$CD25$^-$), DP thymocytes (CD4$^+$CD8$^+$), CD4 SP thymocytes (CD4$^+$CD8$^-$), CD8 SP thymocytes (CD4$^-$CD8$^+$), splenic naïve CD4 T (TCRβ$^+$Thy1.2$^+$CD4$^+$CD8$^-$CD62L$^+$CD44$^-$), splenic naïve CD8 T (TCRβ$^+$Thy1.2$^+$CD4$^-$CD8$^+$CD62L$^+$CD44$^-$), T_{FH} cells (CXCR5$^+$PD-1$^+$CD4$^+$B220$^-$), NK cells (TCRβ$^-$CD49b$^+$) and granulocytes (Gr1$^+$Mac1$^+$). The lineage cocktails contained the following antibodies: anti-TCRβ, anti-CD3, anti-Gr1, anti-CD11b, anti-NK1.1, anti-CD19 and anti-Ly6C for MPPs, ALPs and BLPs, and anti-CD4, anti-CD8, anti-F4/80 and anti-CD21 for plasma cells. Cell populations for *in vitro* stimulation of CD43$^-$ B cells were gated as follows: activated B cells (CD22$^+$CD138$^-$), pre-plasmablast (CD22$^-$CD138$^-$) and plasmablast (CD22$^-$CD138$^+$). Flow cytometry experiments and FACS sorting were performed on LSR Fortessa (BD Biosciences) and FACSAria III (BD Biosciences) machines, respectively. Flowjo software (Treestar) was used for data analysis.

Generation of bone marrow chimeras

For the generation of mixed bone marrow chimeras, donor-derived bone marrow cells of the indicated genotypes (Fig 6) were stained with anti-CD19, anti-CD4, anti-CD8α, anti-TCRβ, anti-TCRγδ, anti-NK1.1 and anti-CD49b antibodies conjugated to PE followed by magnetic depletion of the PE-labeled cells using MACS cell separation (Miltenyi Biotec). The donor cells were mixed at a 1:15 ratio (J_HT: Eβ$^{–/–}$ or Eβ$^{–/–}$ *Prdm1*$^{ihCd2/+}$) and transferred intravenously into lethally irradiated (1,000 rads) *Rag2*$^{–/–}$ recipients. The mice were analyzed 4 months after bone marrow reconstitution.

Injection of apoptotic thymocytes

To generate apoptotic cells, thymocytes of 6-8-week-old C56BL/6 mice were cultured for 6 h at 22 ºC in IMDM medium containing 10% FCS (GE Healthcare; A15-101), 1 mM glutamine, 50 μM β-mercaptoethanol and 1 μM dexamethasone (Sigma-Aldrich), as described (Duhlin et al., 2016). Staining with annexin V and propidium iodide confirmed apoptosis in ~70% of the

thymocytes. Approximately 1 × 10^7 apoptotic thymocytes were intravenously injected into each mouse.

Apoptosis assays

Ex vivo apoptosis of pro-B and pre-B cells was assessed by flow cytometry by using the Violet Ratiometric Membrane Asymmetry Probe/Dead Cell Apoptosis Kit (Thermo Fisher Scientific) according to the manufacturer's instructions. Alternatively, *ex vivo* apoptosis was assessed by intracellular staining with an anti-cleaved Caspase 3 antibody (5A1E; Cell Signaling Technology).

BrdU labeling of B cells

Prdm1$^{ihCd2/+}$ and *Prdm1*$^{+/+}$ mice at the age of 3 months were intraperitoneally injected with 100 μl of 10 mg/ml BrdU (in PBS) at day 0. At the same time, BrdU was added at a concentration of 1 mg/ml to the drinking water, and the BrdU-containing drinking water, which was protected from light, was exchanged every day. At day 10, the mice were either sacrificed or received normal drinking water (without BrdU) for the next 15 days. At day 10 or 25, the BrdU incorporation into immature B and FO B cells of the spleen was analyzed by flow cytometry. Incorporated BrdU was detected by intracellular staining with an anti-BrdU antibody using the APC BrdU Flow kit (BD Pharmingen).

Intracellular staining

Intracellular staining with anti-Blimp1 (5E7), anti-Bcl6 (K112-91) and anti-cleaved Caspase 3 (5A1E) antibodies was performed after fixation and permeabilization of lymphocytes with the Foxp3 staining buffer set (eBioscience).

***In vitro* culture of pro-B cells**

Prdm1$^{ihCd2/+}$ and wild-type pro-B cells were cultured on OP9 stromal cells in IMDM medium containing IL-7 as described (Nutt et al., 1997).

***In vitro* B cell stimulation experiments**

Immature B cells ($CD19^+B220^+IgM^+IgD^-$) were sorted by flow cytometry from the bone marrow, and mature FO B cells were isolated from the spleen by immunomagnetic depletion of CD43-expressing cells using MACS cell separation (Miltenyi Biotec). Mature cells were resuspended in stimulation medium (IMDM medium supplemented with 10% heat-inactivated FCS (GE Healthcare; A15-101), 1 mM glutamine and 50 μM β-mercaptoethanol) and were seeded at a density of 2 × 10^6 cells in 4 ml of stimulation medium into one well of a 6-well plate. Immature B cells were seeded at a density of 1.5 × 10^5 cells in 750 μl of stimulation medium (RPMI 1640 instead of IMDM plus further addition of 1 mM sodium pyruvate and 10 mM Hepes) into one well of a 24-well plate. The stimulation medium additionally contained the following reagents: 25 μg/ml LPS (L4130; Sigma-Aldrich) for LPS stimulation; 3 μM CpG (ODN 1826, InvivoGen) for CpG stimulation; 20 ng/ml IL-4 (made in-house), 10 ng/ml IL-5 (405-ML, R&D Systems) and 2 μg/ml anti-CD40 antibody (HM40-3, eBioscience) for IL-4, IL-5 plus anti-CD40 stimulation. At

60 hours (immature B cells) or 4 days (mature B cells) of stimulation, the relative frequency of $CD138^{+}CD22^{lo}$ plasmablasts was determined by flow cytometric analysis.

Analysis of somatic hypermutation

Following isolation of genomic DNA from sorted splenic plasma cells of non-immunized 4-month-old mice, the intronic region downstream of the J_H4 segment of the *Igh* locus was PCR-amplified with the PfuTurbo DNA polymerase (Agilent) using the PfuUltra II hotstart master mix (Agilent) and primers described in Table EV3. The 564-bp PCR fragments were A-tailed by Taq polymerase (made in-house) and directly cloned using the pGEM-T Easy Vector System (Promega), followed by transformation of the *E. coli* strain DH5α. The inserted DNA of at least 85 clones per genotype was analyzed by Sanger sequencing, and mutations were identified by comparison with the wild-type downstream J_H4 sequence.

Nuclear extract preparation and immunoblot analysis

Nuclear extracts of short-term cultured pro-B cells were prepared as described (Minnich et al., 2016). The protein concentration of the nuclear extract was determined by Bradford assay (BioRad). The proteins of the nuclear extract were denatured in 2× SDS sample buffer, boiled, separated by SDS-PAGE and analyzed by immunoblot analysis.

Immunization, ELISPOT and ELISA analyses

The immune response to a T cell-dependent antigen was studied by intraperitoneal injection of 100 μg of 4-hydroxy-3-nitrophenylacetyl-conjugated keyhole limpet hemocyanin (NP-KLH; Biosearch Technologies) in alum. The frequencies of NP-specific IgM antibody-secreting cells (ASCs) were determined in the spleen by enzyme-linked immunospot (ELISPOT) assay, as described (Smith et al., 1997). NP_{24}-BSA-coated plates were used for capturing total anti-NP-IgM antibodies secreted by individual cells. ASCs were incubated for 6 h at 37 ºC and 5% CO_2, and the resulting spots were visualized with a goat anti-mouse IgM antibody conjugated to alkaline phosphatase (SouthernBiotech), and color was developed by the addition of BCIP/NBT Plus solution (SouthernBiotech). After extensive washing, the spots were counted with an AID ELISPOT reader system (Autoimmun Diagnostika).

The serum titer of NP-specific IgM, IgG1 and IgG2b antibodies was determined by enzyme-linked immunosorbant assay (ELISA) (Smith et al., 1997) by using ELISA plates (Sigma-Aldrich), which were coated with 25 μg/ml of NP_7-BSA or NP_{24}-BSA to capture high-affinity IgG1 or total NP-specific IgM, IgG1 and IgG2b antibodies, respectively. The serum concentration of NP-specific IgG1 was determined relative to that of a standard anti-NP IgG1 antibody (hybridoma SSX2.1).

ELISA measurements of autoantibodies

ELISA plates (Sigma-Aldrich), which were coated with mouse liver DNA and then blocked with 1% BSA, were incubated with mouse serum for 2 h at 22 ºC. Anti-DNA-specific antibodies were detected by incubation with horseradish peroxidase-conjugated goat anti-mouse IgG or goat anti-

mouse IgM antibodies (both from SouthernBiotech) in the presence of the TMB substrate (Biolegend). The absorbance was measured at 650 nm using an Epoch Microplate Spectrophotometer (BioTek Instruments). Antibodies against cardiolipin were measured as described (Wermeling et al., 2010). Briefly, ELISA plates were coated with cardiolipin (Sigma-Aldrich) overnight. Serum was added after blocking, and antigen-reactive IgG and IgM were measured with alkaline phosphate- or horseradish peroxidase-conjugated anti-mouse antibodies (SouthernBiotech). Antibodies against SSA (Ro-52) and SSB (La) were measured using commercial kits (all from Signosis Inc) following the manufacturer's instruction. All samples were corrected for background binding.

Indirect immunofluorescence assay using HEp-2 slides

Diluted mouse serum (1:100 in PBS) was incubated on HEp-2 slides (Orgentech) for 30 min in the dark at 22 ºC using a humidity chamber. Subsequently, the slides were rinsed once with a squirt bottle and washed twice for 5 min with PBS. For detection of mouse IgM or IgG, the slides were incubated for 30 min in the dark at 22 ºC in a humidity chamber with an Alexa488-conjugated goat anti-mouse IgM antibody or an Alexa488-conjugated goat anti-mouse IgG (H+L) antibody (both from Thermo Fisher Scientific and diluted in PBS) as a secondary antibody. Following two washing steps, DAPI-containing mounting medium (Life Technologies) was added, and images were acquired with a Zeiss Axio Imager 2 microscope and were analyzed with the Fiji software.

Detection of IgG immune complexes

IgG immune complexes were detected on paraformaldehyde-fixed cryosections of kidneys by staining with an Alexa488-conjugared goat anti-mouse IgG (H+L) antibody (Thermo Fisher Scientific) followed by addition of DAPI-containing mounting medium (Life Technologies). The abundance of IgG immune complexes was quantified by determining the mean fluorescence intensity (MFI) of at least 12 individual glomeruli for each kidney. Image acquisition was performed with a LSM710 (Zeiss) confocal microscope, and images were analyzed with the Fiji software.

Histopathological analysis

For histopathological analyses, one kidney from each mouse was isolated, fixed in 4% paraformaldehyde, trimmed, dehydrated and processed with the Logos Tissue processor. Processed kidneys were embedded in paraffin, sectioned at a thickness of 2 μm and stained either with haematoxylin and eosin (H&E) or periodic acid-Schiff (PAS) stains. Stained slides were reviewed by a board-certified pathologist with a Zeiss Axioskop 2 MOT microscope (Carl Zeiss Microscopy) and representative microscopic images were acquired with a SPOT Insight digital camera (SPOT Imaging). From each kidney, 40 glomeruli were individually evaluated based on criteria adapted from the following references (Alperovich et al., 2007; Chowdhary et al., 2015; Weening et al., 2004). Each glomerulus was evaluated for the presence of the following microscopic lesions: active lesions - mesangial hypercellularity, fibrinoid necrosis; endocapillary hypercellularity, capillary basement membrane lesions (including wireloops, reduplication and

focal granular sub- or supra- basement membrane deposits) and intracapillary hyaline thrombi as well as chronic lesions - mesangial sclerosis, mesangial crescents and capsular fibrous adhesions. Light microscopic lesions that were notably evident in kidneys from female *Prdm1*$^{ihCd2/+}$ mice included capillary basement membrane lesions (focal granular deposits, focal or segmental basement membrane thickening or reduplication) and mesangial sclerosis (expansion of the mesangium with PAS-positive matrix with narrowing of capillary lumina). Intracapillary hyaline thrombi were evident in a few mice as were obsolescent glomeruli. Tubular and interstitial lesions were not assessed, as they were not a prominent feature in any of the kidneys. Histopathologic scores were assigned to each glomerulus, based on the extent of involvement by one or more of the above lesions as follows: score 0 - within normal limits; score 1 - minimal (less than 10%); score 2 - mild, segmental (11% to 30%); score 3 - moderate, segmental (31% to 60%); score 4 - severe, segmental to global (greater than 60%), as documented in Table EV2.

RT-qPCR analysis of nascent transcripts and spliced mRNA

Total RNA was isolated from sorted pro-B cells, pre-B cells and *in vitro* LPS-stimulated plasmablasts by using the RNeasy Mini kit (Qiagen). Genomic DNA was eliminated by using an eliminator spin column (Qiagen). Reverse transcription was performed by using randon hexamer or oligo-dT primers (New England Biolabs) and SuperScript II reverse transcriptase (Life Technologies). *Prdm1*, *Atg5* and *Tbp* nascent transcripts were analyzed by PCR amplification with primers located in intronic sequences (Table EV3), and the data were normalized to those obtained for nascent *Tbp* transcripts. The *Prdm1* and *Tbp* mRNAs were analyzed by PCR amplification with primers located in different exons (Table EV3), and the data were normalized to the *Tbp* mRNA.

GRO-seq analysis

CD19^{+} pro-B cells from the bone marrow of *Rag2*$^{-/-}$ mice and CD23^{+} FO B cell from the spleen of *Cd23*-Cre *Ebf1*$^{fl/+}$ or *Cd23*-Cre *Ebf1*$^{fl/-}$ mice were isolated by immunomagnetic enrichment using MACS cell separation (Miltenyi Biotec). The nuclei were prepared from approximately 10 million cells by incubation with nuclear preparation buffer (0.30 M sucrose, 10 mM Tris, pH 7.5, 60 mM KCl, 15 mM NaCl, 5 mM MgCl2, 0.1 mM EGTA, 0.1% NP40, 0.15 mM spermine, 0.5 mM spermidine and 2 mM 6AA) for 3 min and were then subjected to nuclear run-on for 5 min at 30 ˚C using BrUTP-containing NTPs, as described (Core et al., 2008). The reaction was stopped by the addition of TRIzol reagent. The RNA was isolated, fragmented and the nascent transcripts were isolated using anti-BrdU antibody-conjugated agarose beads (Santa Cruz Biotech, sc-32323-ac). The purified nascent RNA was subjected to the small RNA library preparation procedure (Reimão-Pinto et al., 2015) with anti-BrdU antibody-mediated purification following each ligation step.

ChIP-qPCR analysis of histone modifications

Short-term cultured pro-B cells were used for ChIP analysis with an anti-H3K4me1 antibody (rabbit polyclonal; Abcam; ab8895) or an anti-H3K27ac antibody (rabbit polyclonal; Abcam;

ab4729), as described (Schebesta et al., 2007). Different regions of the *Prdm1* locus were analyzed by ChIP-qPCR analysis with specific primers (shown in Table EV3), as described (Minnich et al., 2016).

ChIP-seq analysis of Blimp1 binding

Chromatin of 1 × 10^8 *in vitro* cultured pro-B cells from *Prdm1*$^{ihCd2/+}$ mice was prepared using a lysis buffer containing 0.25% SDS and was then subjected to ChIP with anti-V5 agarose beads (Sigma-Aldrich), as described (Schebesta et al., 2007). The quantification of precipitated DNA was performed using qPCR, and about 1-5 ng of ChIP-precipitated DNA was used for library preparation and subsequent Illumina deep sequencing (Table EV4).

cDNA preparation for RNA-seq

Total RNA from *ex vivo* sorted pro-B and pre-B cells was isolated with the RNeasy Plus Mini Kit (Qiagen), and mRNA was purified by two rounds of poly(A) selection with the Dynabeads mRNA purification kit (Invitrogen). The mRNA was fragmented by heating at 94 °C for 3 min in fragmentation buffer. The fragmented mRNA was used as template for first-strand cDNA synthesis with random hexamers and the Superscript Vilo First-Strand Synthesis System (Invitrogen). The second-strand cDNA synthesis was performed with 100 mM dATP, dCTP, dGTP and dUTP in the presence of RNase H, *E. coli* DNA polymerase I and DNA ligase (Invitrogen). The incorporation of dUTP allowed for specific elimination of the second DNA strand during library preparation, thereby preserving strand specificity (Parkhomchuk et al., 2009).

Library preparation and Illumina Deep Sequencing

About 1-5 ng of cDNA or ChIP-precipitated DNA was used as starting material for the generation of sequencing libraries with the NEBNext Ultra Ligation Module and NEBNext End Repair/dA-Tailing module. DNA fragments of the following sizes were selected: 200–500 bp for ChIP-seq and 150–700 bp for RNA-seq with AMPure XP beads (Beckman Coulter). For strand-specific RNA-seq, the uridines present in one cDNA strand were digested with uracil-N-glycosylase (New England BioLabs) as described (Parkhomchuk et al., 2009), followed by PCR amplification with the KAPA Real Time Amplification kit (KAPA Biosystems). Completed libraries were quantified with the Bioanalyzer dsDNA 1000 assay kit (Agilent) and QPCR NGS Library Quantification kit (Agilent). Cluster generation and sequencing was carried out by using the Illumina HiSeq 2000 system with 50 nucleotides read length according to the manufacturer's guidelines.

Database of RefSeq-annotated genes

The database generation of RefSeq-annotated genes was performed as previously described (Wöhner et al., 2016). To refine the annotation of immunoglobulin genes, the immunoglobulin λ light-chain segments were replaced with their corresponding converted GRCm38.p3 annotations (Ensembl version 79; Yates et al., 2016). The resulting number of genes was 24,732.

Sequence alignment

In case of RNA-seq experiments, reads corresponding to mouse ribosomal RNAs (BK000964.1 and NR046144.1) were removed. The remaining reads were cut down to a read length of 44 nucleotides and aligned to the mouse transcriptome (genome assembly version of July 2007 NCBI37/mm9) using TopHat version 1.4.1 (Trapnell et al., 2009). In case of ChIP-seq, GRO-seq and ATAC-seq experiments, all sequence reads that passed the Illumina quality filtering were considered for alignment after adapter trimming. The remaining reads were aligned to the mouse genome assembly version of July 2007 (NCBI37/mm9), using the Bowtie program versions 0.12.1, 1.0.0 and 2.1.0, respectively (Langmead et al., 2009). For GRO-seq, additional four bases were eliminated after adapter trimming and filtered against the rDNA with Bowtie version 2.1.0 before mouse genome alignment. For ATAC-seq, additional alignment parameters were '-sensitive -X 5000'.

Peak calling

Blimp1 peaks were called using the MACS program version 2.1.0 (Zhang et al., 2008) with default parameters and appropriate input control for pro-B and mature B cells [pro-B cells - GSM1145867 (Schwickert et al., 2014); mature B cells - GSM2058441, (Wöhner et al., 2016)] and a genome size of 2.654.911.517 bp (mm9). Peak calling identified 889 Blimp1 peaks in *Prdm1*$^{ihCd2/+}$ pro-B cells and 14,512 Blimp1 peaks in *Prdm1*$^{Bio/Bio}$ plasmablasts with a *P* value of $< 10^{-5}$. These Blimp1 peaks were further filtered for a *P* value of $< 10^{-10}$, which resulted in 762 peaks in pro-B cells and 9,320 peaks in plasmablasts.

Peak overlap analysis

The peak overlap analysis was performed with the Multovl program (Aszódi, 2012) by using a minimal overlap length of one bp and allowing for all possible overlaps.

Motif discovery analysis

Sequences +/- 150 bp around the most significant MACS2 summit of the top 300 *P* value-ranked Blimp1 peaks have been used as input for the MEME-ChIP suite version 4.9.1 (Machanick and Bailey, 2011). The most significant motif was the Bimp1 motif with an E-value of 1.3×10^{-379}.

Read density analysis

Read density profiles were calculated using jnomics (I. Tamir, unpublished).

Peak-to-gene assignment

Common and unique Blimp1 targets in *Prdm1*$^{ihCd2/+}$ pro-B cells and *Prdm1*$^{Bio/Bio}$ plasmablasts were identified by peak-to-gene assignment as described (Revilla-i-Domingo et al., 2012). Peaks were assigned to genes in a stepwise manner by prioritizing genes containing peaks in their promoter and/or gene body. For this, peaks overlapping with the promoter (-2.5 kb to +2.5 kb relative to TSS) or gene body (+2.5 kb to TES) were first assigned to the corresponding gene. Other peaks within a specified region of 50 kb upstream of the TSS or downstream of the TES

were assigned to the gene containing peaks in the promoter or gene body. All other peaks within the same specified region were assigned to the nearest gene, and all non-assigned peaks were classified as intergenic.

Analysis of RNA-seq data

The number of reads per gene was counted using featureCounts version 1.5.0 (Liao et al., 2014) with default settings. TPM (transcripts per million) values were calculated as described (Wagner et al., 2012). For analysis of differential gene expression of *Prdm1*$^{ihCd2/+}$ and wild-type pro-B and pre-B cells, the datasets were grouped according to cell type, genotype and replicate number and were analyzed using the R package DESeq2 version 1.8.2 (Love et al., 2014). Wald tests were performed with the model design formula "~ replicate + type" (type is a linear combination of cell type and genotype). Sample normalizations and dispersion estimations were conducted using the default DESeq2 settings. Regularized log transformations were computed with the blind option set to 'FALSE' and were transformed from $\log_2$ to $\log_{10}$ scale for the scatterplots shown in Fig 4A,B. Genes with an adjusted P value < 0.05 and an absolute fold change > 3 as well as a mean TPM (averaged within conditions) > 5 were called as significantly expressed. Immunoglobulin and T cell receptor genes were filtered from the list of significantly expressed genes, but were included in the TPM calculations.

Statistical analysis

Statistical analysis was performed with the GraphPad Prism 7 software. The two-tailed Student's *t*-test analysis was used to assess the statistical significance of differences between two experimental groups in all experiments, with the exception of those involving NGS-based approaches and ELISA measurement. The statistical evaluation of the RNA-seq data is described above (Analysis of RNA-seq data). The ELISA data were analyzed using the Mann-Whitney test.

Data availability

RNA-seq, ChIP-seq and GRO-seq data (Table EV4), which are first reported in this study, are available at the Gene Expression Omnibus (GEO) repository under the accession numbers GSE111692. Previously published ATAC-seq, ChIP-seq and RNA-seq datasets, which were used in this study, are available at the GEO repository under the accession numbers indicated in Table EV4.

2. Appendix Figures

Appendix Figure S1

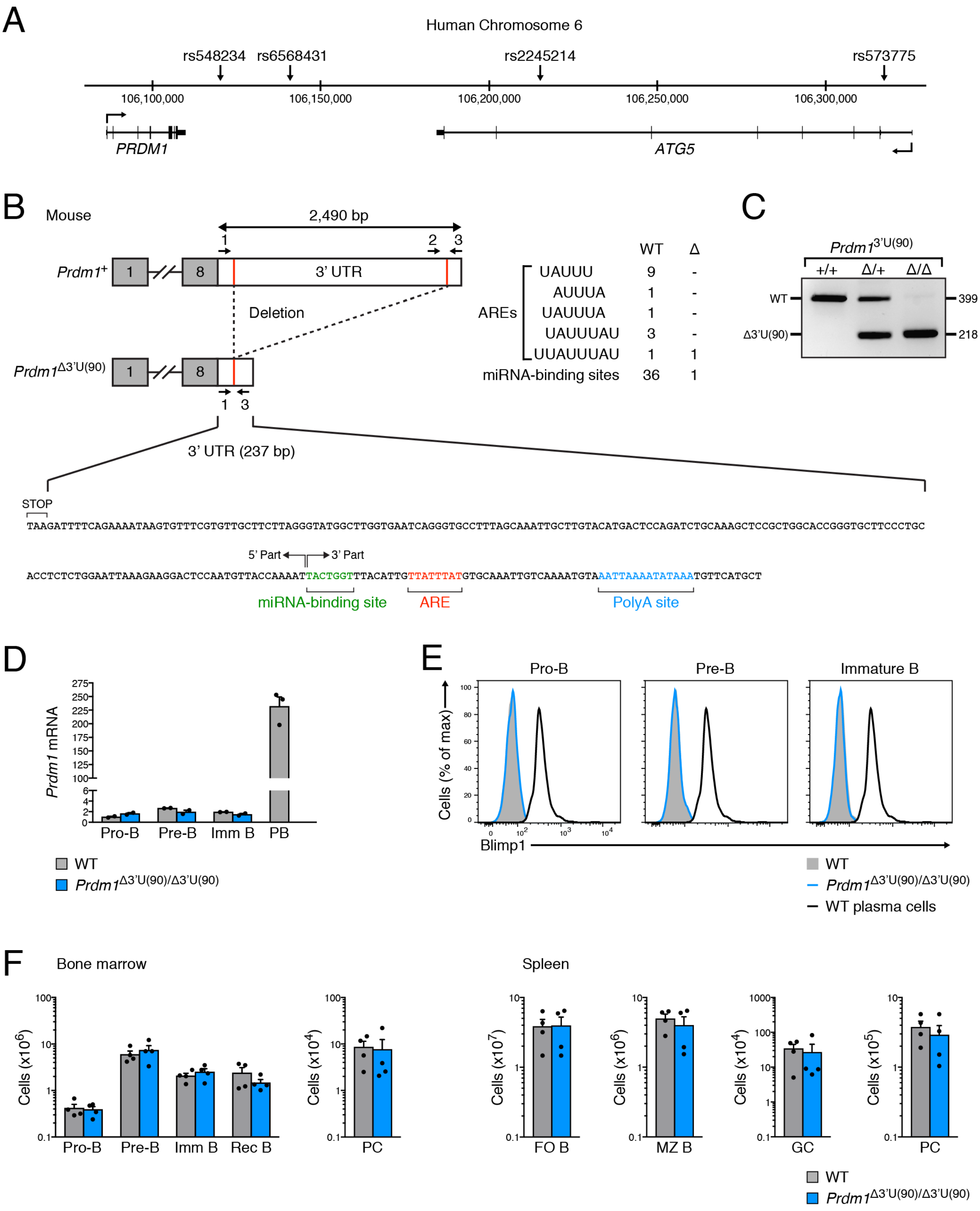

Appendix Figure S1. Normal posttranscriptional control of Blimp1 expression in *Prdm1*$^{\Delta3'U(90)/\Delta3'U(90)}$ mice.

(**A**) Schematic diagram of the human *ATG5-PRDM1* region. The exon-intron structures of both genes are shown together with the positions of the two SNPs (rs6568431 and rs548234) that have been associated with human SLE and RA (Gateva et al., 2009; Raychaudhuri et al., 2009; Zhou et al., 2011). The hg38 genomic coordinates of human chromosome 12 are shown. (**B**) Schematic diagram of the *Prdm1*$^{\Delta3'U(90)}$ allele. A 2,253-bp sequence containing most AU-rich elements (AREs) and predicted microRNA-binding sites (http://www.mirdb.org) was deleted from the 3' UTR (2,490 bp) of the *Prdm1* gene by CRISPR/Cas9-mediated mutagenesis. The truncated 3' UTR sequence of the *Prdm1*$^{\Delta3'U(90)}$ allele is shown together with the stop codon, polyadenylation (polyA) motif, residual ARE sequence and remaining microRNA-binding site (predicted to bind miR-470). The positions of the genotyping primers (1-3) are indicated. (**C**) PCR genotyping of DNA isolated from *Prdm1*$^{\Delta3'U(90)/\Delta3'U(90)}$, *Prdm1*$^{\Delta3'U(90)/+}$ and *Prdm1*$^{+/+}$ mice. The PCR fragments corresponding to the wild-type (WT) and *Prdm1*$^{\Delta3'U(90)}$ alleles are indicated to the left and their size (base pairs) to the right of the gel. (**D**) No increase of mature *Prdm1* mRNA in *Prdm1*$^{\Delta3'U(90)/\Delta3'U(90)}$ B cells. *Prdm1* mRNA levels were determined by RT-qPCR analysis in *ex vivo* sorted pro-B, pre-B and immature B cells from the bone marrow of wild-type (WT, gray) and *Prdm1*$^{\Delta3'U(90)/\Delta3'U(90)}$ (blue) mice. Sorted wild-type plasmablasts (PB, gray) generated by LPS stimulation for 4 days were used as a control. The value measured for the *Prdm1* mRNA was normalized to the corresponding value of the *Tbp* mRNA coding for the ubiquitous TATA box-binding protein. The *Prdm1* mRNA data are shown as mean value with SEM. The primers used for PCR amplification are shown in Table EV3. (**E**) Absence of Blimp1 expression in *Prdm1*$^{\Delta3'U(90)/\Delta3'U(90)}$ B cells. Blimp1 protein levels were determined by intracellular staining in bone marrow pro-B, pre-B and immature B cells from wild-type (WT, gray) and *Prdm1*$^{\Delta3'U(90)/\Delta3'U(90)}$ (blue line) mice. The intracellular staining profile of wild-type splenic plasma cells (black line) is shown for comparison. (**F**) Normal B cell development in *Prdm1*$^{\Delta3'U(90)/\Delta3'U(90)}$ mice. Absolute cell numbers of the indicated B cell types were determined by flow cytometric analysis of bone marrow and splenic B cells from wild-type (WT, gray), and *Prdm1*$^{\Delta3'U(90)/\Delta3'U(90)}$ (blue) mice. Bar graphs show the statistical data as mean value with SEM. Each dot corresponds to one mouse.

Appendix Figure S2

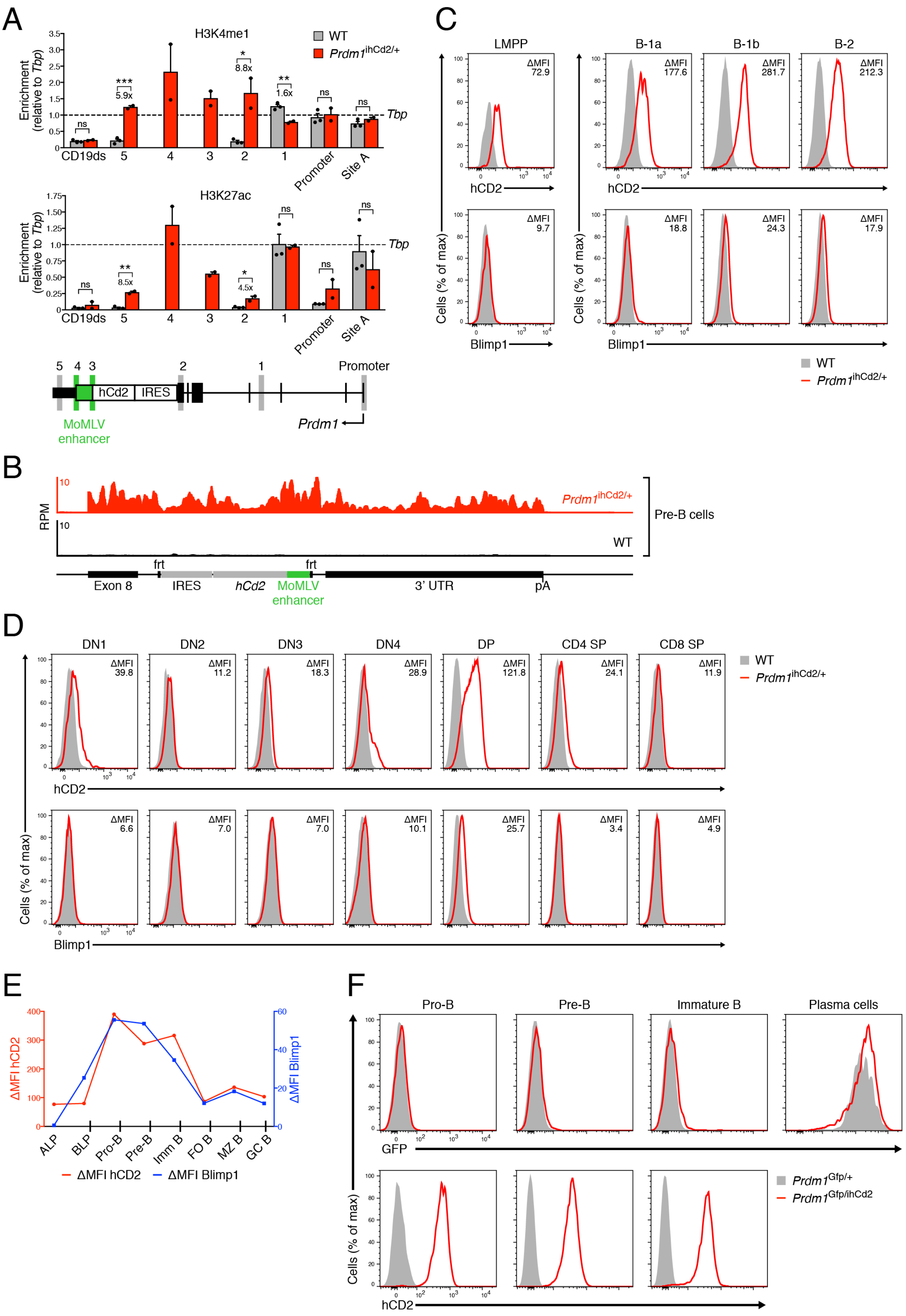

Appendix Figure S2. Ectopic Blimp1 expression in different lymphocyte subsets of *Prdm1*$^{ihCd2/+}$ mice.

(**A**) Induction of active chromatin at the 3' end of the *Prdm1*^{ihCd2} gene by the inserted MoMLV enhancer. ChIP analysis with H3K4me1- or H3K27ac-specific antibodies was used to determine the abundance of active chromatin (H3K4me1 and H3K27ac) at 7 different regions of the *Prdm1* locus in short-term cultured wild-type (WT) and *Prdm1*$^{ihCd2/+}$ pro-B cells. Input and precipitated DNA were quantified by qPCR with primers amplifying the indicated regions, shown below a schematic diagram of the *Prdm1* gene, or the promoter of the ubiquitously expressed control *Tbp* gene. The amount of precipitated DNA was determined as percentage relative to input DNA for each region analyzed and is shown as relative enrichment at the *Prdm1* region compared to the *Tbp* promoter (set as 1). Average values with SEM are shown for two independent experiments. An inactive region downstream of the *Cd19* gene (Cd19ds) was analyzed as negative control. Site A corresponds to the upstream region A shown in Fig 1A. The amplicons 3 and 4 (green) could only be amplified from the MoMLV-containing *Prdm1*^{ihCd2} allele. (**B**) Inclusion of the *Prdm1* 3' UTR sequence in the *Prdm1-ihCd2* transcript, as shown by the presence of RNA-seq reads throughout the last exon 8 of the *Prdm1-ihCd2* mRNA in *Prdm1*$^{ihCd2/+}$ pre-B cells in contrast to the absence of reads in wild-type pre-B cells. (**C**,**D**) Flow cytometric analysis of hCD2 expression (top row) and intracellular Blimp1 staining (bottom row) in LMPPs from the bone marrow as well as in B-1a, B-1b and B-2 cells from the peritoneal cavity (**C**) and in all thymocyte subsets (**D**) of wild-type (gray) and *Prdm1*$^{ihCd2/+}$ (red) mice. The difference in mean fluorescence intensity (ΔMFI) between the two genotypes is shown for each cell type. (**E**) Correlation plot of the ΔMFI values determined for hCD2 and Blimp1 expression at the indicated B cell developmental stages. **(F)** Flow cytometric analysis of GFP (top row) and hCD2 (bottom row) expression in bone marrow pro-B, pre-B and immature B cells as well as in splenic plasma cells of *Prdm1*$^{Gfp/+}$ (gray) and *Prdm1*$^{Gfp/ihCd2}$ (red) mice.

Appendix Figure S3

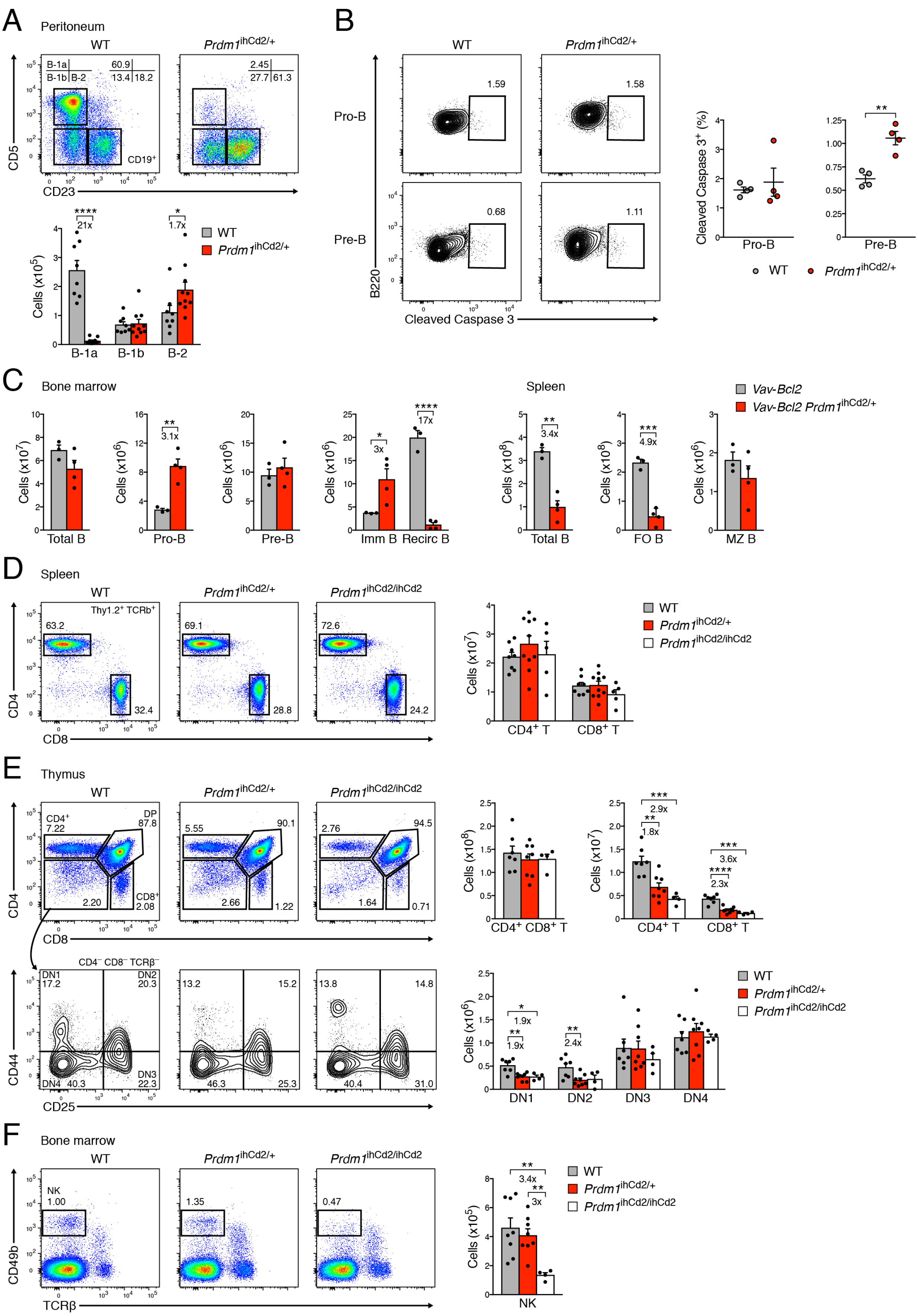

Appendix Figure S3. **Phenotypic analysis of immune cells in *Prdm1*$^{\mathbf{ihCd2/+}}$ mice.**

(**A**) Flow cytometric analysis of B-1a, B-1b and B-2 cells from the peritoneal cavity of *Prdm1*$^{ihCd2/+}$ (red) and wild-type (WT, gray) mice. Bar graphs indicate absolute cell numbers for the indicated cell types. (**B**) Flow cytometric analysis of apoptotic pro-B and pre-B cells from the bone marrow of wild-type and *Prdm1*$^{ihCd2/+}$ mice, as determined by intracellular staining of cleaved Caspase 3. Dot plots (to the right) show the relative frequency of cleaved Caspase 3-positive pro-B and pre-B cells for both genotypes. **(C)** Flow cytometric analysis of the indicated B cell types from the bone marrow or spleen of *Vav-Bcl2 Prdm1*$^{ihCd2/+}$ and *Vav-Bcl2* mice at the age of 2 months. Bar graphs show absolute cell numbers for each cell type and indicated genotype. **(D-F)** Flow cytometric analysis of splenic CD4 T and CD8 T cells (**D**), thymic T cell subsets (**E**) and bone marrow NK cells (**F**) from wild-type (gray), *Prdm1*$^{ihCd2/+}$ (red) and *Prdm1*$^{ihCd2/ihCd2}$ (white) mice. Bar graphs show absolute cell numbers for each cell type and indicated genotype. The different cell types were defined as described in detail in the Appendix Supplementary Methods. Statistical data (**A-F**) are shown as mean value with SEM and were analyzed by the Student's *t*-test; *$P < 0.05$, **$P < 0.01$, ***$P < 0.001$, ****$P < 0.0001$. Each dot corresponds to one mouse.

Appendix Figure S4

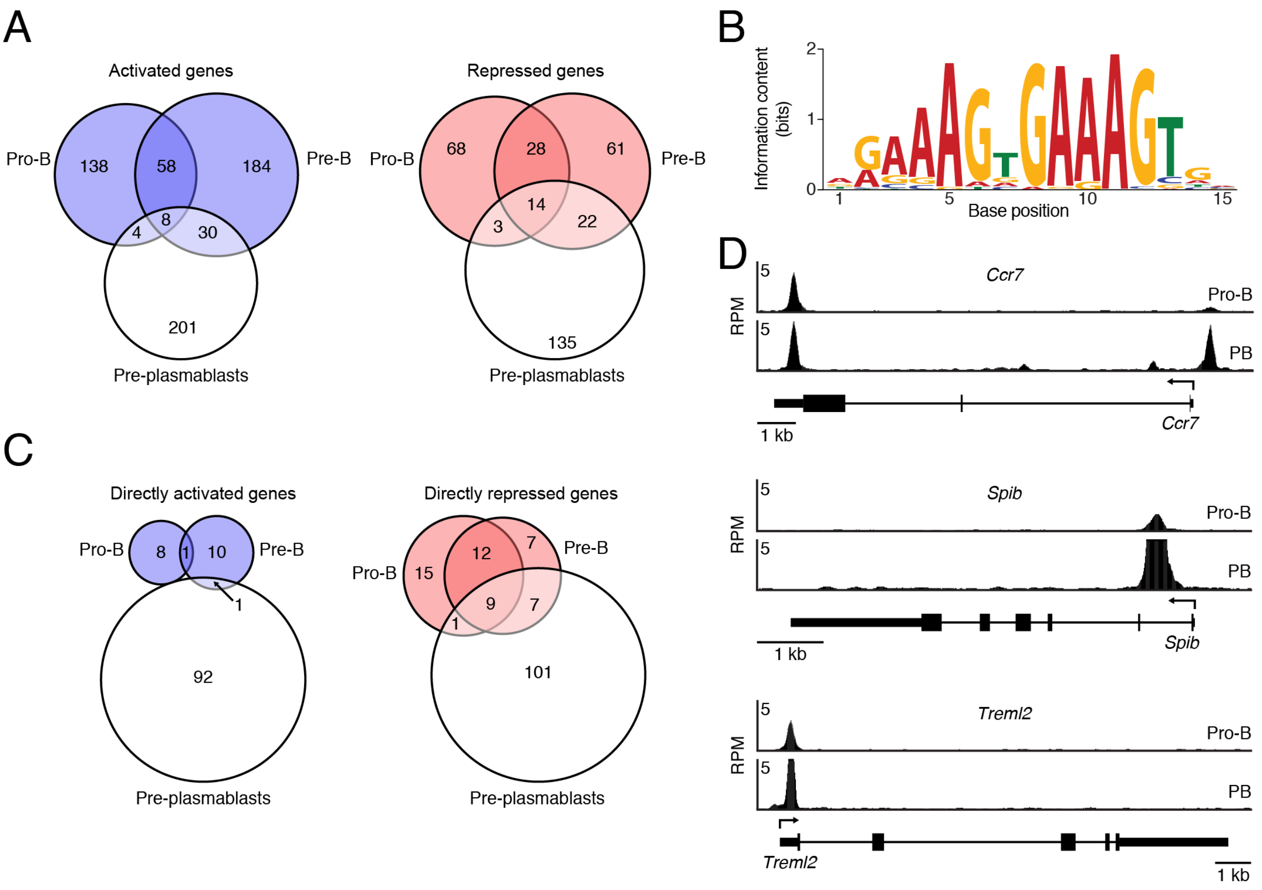

E Blimp1-regulated genes in pre-B cells

Gene function	Activated (bound)	Repressed (bound)
Surface receptors	45 (4)	23 (8)
Signal transducers	51 (4)	18 (2)
Transcription regulators	24 (0)	10 (4)
Metabolism	21 (0)	13 (5)
Transporters	13 (0)	4 (0)
Secreted proteins	11 (1)	7 (3)
Cytoskeleton	11 (1)	7 (1)
Protein turnover	7 (1)	4 (1)
ER function	5 (0)	2 (1)
Protein trafficking	3 (0)	3 (2)
Cell cycle	3 (0)	2 (2)
Other functions	44 (1)	13 (2)
Unknown functions	42 (0)	19 (4)
Total genes	280 (12)	125 (35)

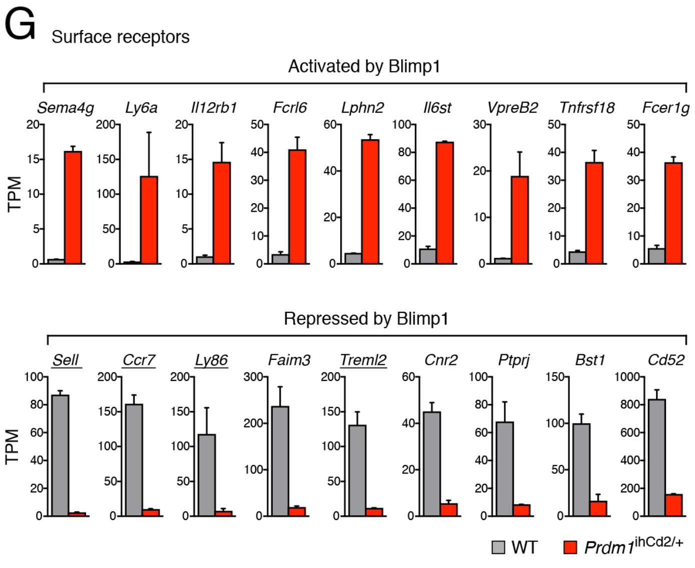

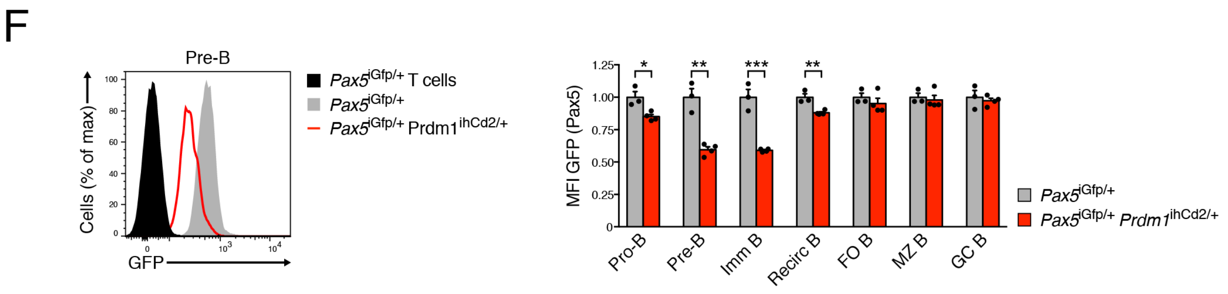

Appendix Figure S4. **Blimp1-dependent deregulation of the B cell gene expression program.** Multiple overlap of Blimp1-activated (left) or Blimp1-repessed (right) genes, which were identified in *Prdm1*$^{ihCd2/+}$ pro-B and pre-B cells (as described in Fig 4A,B) as well as in wild-type pre-plasmablasts (as described in Fig 1g; Minnich et al., 2016). The number of genes in each sector of the Venn diagram is indicated. (**B**) Consensus Blimp1-binding motif, which was identified by *de novo* motif discovery in Blimp1 peaks of *Prdm1*$^{ihCd2/+}$ pro-B cells with an E-values of 1.3 x 10^{-379}. (**C**) Venn diagrams indicating the overlap of activated Blimp1-bound (left) and repressed Blimp1-bound (right) target genes, which were determined in *Prdm1*$^{ihCd2/+}$ pro-B and pre-B cells (as described in Fig 4F) as well as in wild-type pre-plasmablasts (as indicated in Fig 2c; Minnich et al., 2016). (**D**) Blimp1 binding at the commonly repressed target genes *Ccr7*, *Spib* and *Treml2*. The ChIP-seq data (left) were obtained with *Prdm1*$^{ihCd2/+}$ pro-B cells and *Prdm1*$^{Bio/Bio}$ *Rosa26*$^{BirA/BirA}$ plasmablasts (PB; Minnich et al., 2016). (**E**) Functional classification and quantification of the proteins that are encoded by the Blimp1-activated and Blimp1-repressed genes identified in *Prdm1*$^{ihCd2/+}$ pre-B cells (Fig 4B). Numbers in brackets indicate genes with Blimp1 peaks in *Prdm1*$^{ihCd2/+}$ pro-B cell. (**F**) Blimp1-mediated repression of *Pax5* during B cell development in *Prdm1*$^{ihCd2/+}$ mice. GFP (Pax5) expression was analyzed by flow cytometry of different B cells subsets from the bone marrow and spleen of *Pax5*$^{iGfp/+}$ *Prdm1*$^{ihCd2/+}$ mice (red), which report *Pax5* (GFP) mRNA expression from an IRES-*Gfp* gene inserted in the 3' UTR of *Pax5* (Fuxa and Busslinger, 2007). Different B cell subsets (gray) and T cells (black) of *Pax5*$^{iGfp/+}$ mice were analyzed as positive or negative control for Pax5 expression. GFP expression is shown as a histogram for pre-B cells (left) or as bar graphs (right) for all B cell subsets relative to the GFP expression determined for the control *Pax5*$^{iGfp/+}$ genotype (set as 1). (**G**) Expression of selected Blimp1-activated and Blimp1-repressed genes coding for cell surface receptors. Blimp1-bound genes are underlined. The mRNA expression of the indicated genes is shown as mean expression value (TPM) with SEM, based on two different RNA-seq experiments for the pre-B cells of each genotype.

Appendix Figure S5

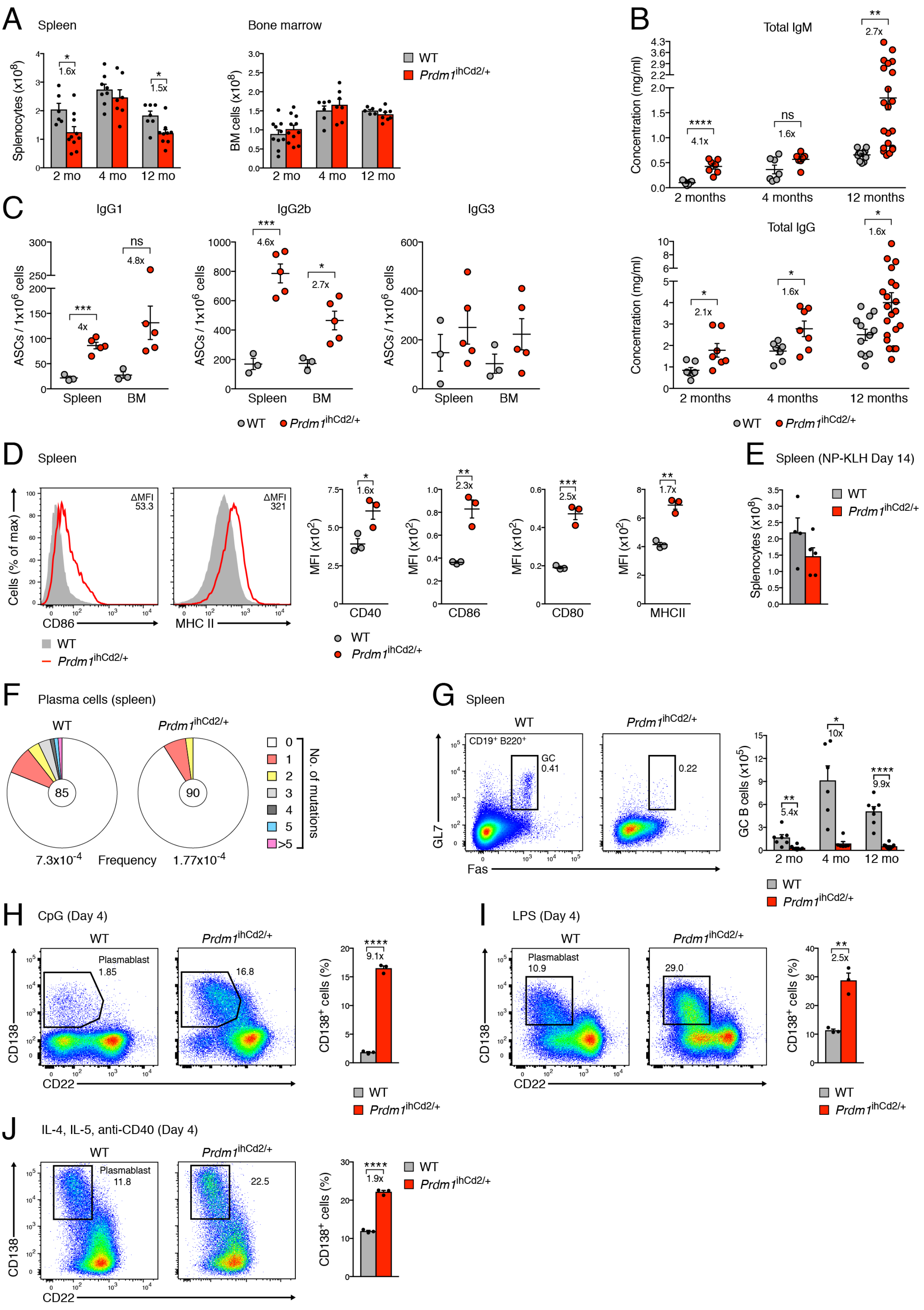

Appendix Figure S5. Impaired GC B cell formation and increased plasmablast differentiation in *Prdm1*$^{ihCd2/+}$ mice.

(**A**) The number of total cells in the spleen (left) and bone marrow (bone marrow) of non-immunized *Prdm1*$^{ihCd2/+}$ (red) and wild-type (WT, gray) mice at the age of 2, 4 and 12 months was determined by flow cytometry. These absolute cell numbers correspond to the data shown in Fig 5A (spleen) and Fig 5B (bone marrow). (**B**) Presence of IgM and IgG antibodies in the serum of non-immunized *Prdm1*$^{ihCd2/+}$ (red dots) and wild-type (gray dots) mice at the age of 2, 4 and 12 months. The titers (mg/ml) of total IgM and IgG antibodies were determined by ELISA and correspond to the mice analyzed in Fig 5A,B. (**C**) Secretion of the indicated IgG isotypes by antibody-secreting cells (ASCs) in the spleen or bone marrow (BM) of non-immunized *Prdm1*$^{ihCd2/+}$ (red dots) and wild-type (gray dots) mice at the age of 7 months, as determined by ELISPOT assay. (**D**) Expression of the activation markers MHCII, CD40, CD80 and CD86 by splenic FO B cells of non-immunized *Prdm1*$^{ihCd2/+}$ (red) and wild-type (WT, gray) mice at the age of 6 weeks, as analyzed by flow cytometry. Dot plots indicate the mean fluorescence intensity (MFI) determined for the FO B cells of both genotypes. (**E**) Number of total splenocytes in 2-month-old *Prdm1*$^{ihCd2/+}$ (red) and wild-type (WT, gray) mice at day 14 after immunization with NP-KLH (in alum), as determined by flow cytometry. These data correspond to the immunization experiment shown in Fig 5C. (**F**) Somatic hypermutation (SHM) frequency of *ex vivo* sorted plasma cells from the spleen of 4-month-old non-immunized *Prdm1*$^{ihCd2/+}$ and wild-type mice, as determined by sequencing of the region downstream of the J_H4 segment of the *Igh* gene. The pie-charts indicate the numbers of analyzed sequences with their corresponding mutations and overall mutation rate determined for plasma cells of each genotype (2 mice analyzed per genotype). (**G**) Flow cytometric analysis of GC B cells from the spleen of non-immunized *Prdm1*$^{ihCd2/+}$ (red) or wild-type (gray) mice. The flow cytometry plots show the analysis of 12-month-old mice. Bar graphs show absolute numbers of GC B cells in the spleen of mice at the age of 2, 4 or 12 months. (**H**-**J**) *In vitro* plasmablast differentiation of FO B cells. $CD43^-$ FO B cells, which were MACS-sorted from the spleen of *Prdm1*$^{ihCd2/+}$ (red) or wild-type (gray) mice, were stimulated for 4 days with either CpG oligodeoxynucleotides (**H**), LPS (**I**) or IL-4, IL-5 and anti-CD40 (**J**), and the relative abundance of $CD138^+CD22^{lo}$ plasmablasts (PB) was determined by flow cytometry at day 4. Statistical data (**A**-**E**, **G**-**J**) are shown as mean value with SEM and were analyzed by the Student's *t*-test; *$P < 0.05$, **$P < 0.01$, ***$P < 0.001$,

Appendix Figure S6

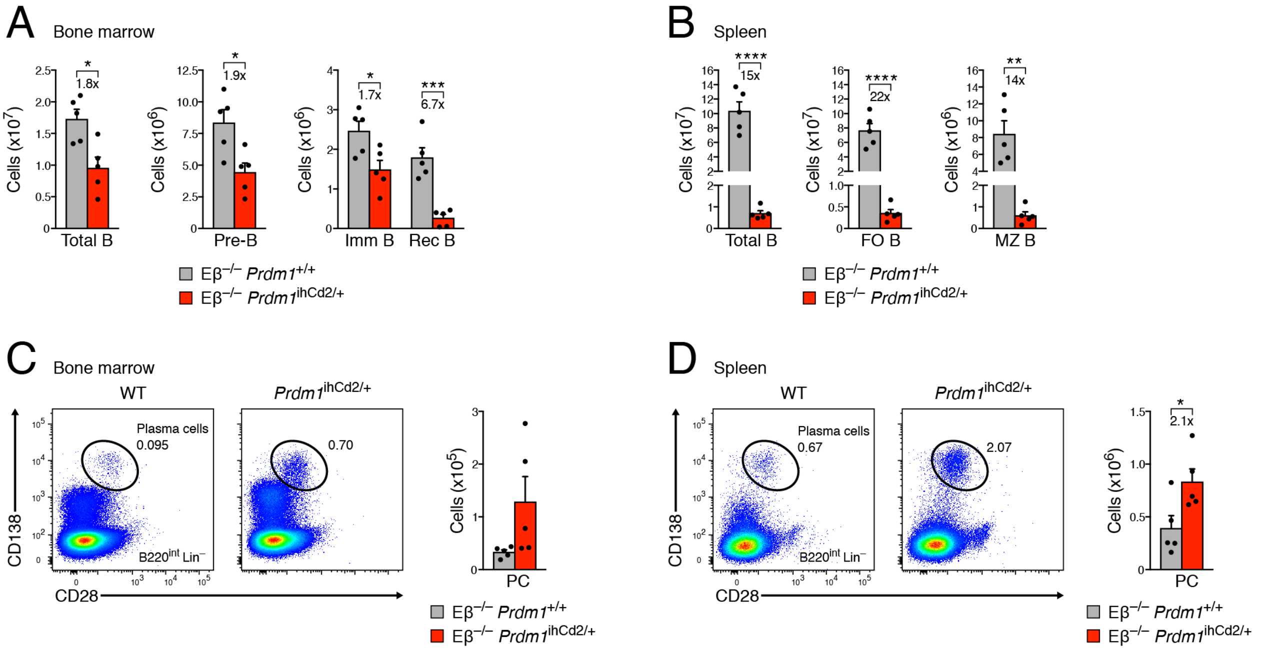

Appendix Figure S6. Decreased B cell numbers and increased plasma cells in Eβ$^{-/-}$ *Prdm1*$^{ihCd2/+}$ mice.

(**A**,**B**) Loss of the different B cell subsets in the bone marrow (**A**) and spleen (**B**) of the T cell-deficient Eβ$^{-/-}$ *Prdm1*$^{ihCd2/+}$ (red) mice relative to the control Eβ$^{-/-}$ *Prdm1*$^{+/+}$ (gray) mice. Bar graphs indicate absolute numbers of the different B cell types, which were analyzed by flow cytometry. (**C**,**D**) Increased plasma cell numbers in Eβ$^{-/-}$ *Prdm1*$^{ihCd2/+}$ (red) mice compared to control Eβ$^{-/-}$ *Prdm1*$^{+/+}$ (gray) mice. Flow cytometric analysis of plasma cells from the bone marrow (**C**) and spleen (**D**) of the indicated genotypes is shown to the left, and bar graphs indicate the absolute cell numbers of plasma cells in each organ to the right. Statistical data (**A-D**) are shown as mean value with SEM and were analyzed by the Student's *t*-test; *$P < 0.05$, **$P < 0.01$, ***$P < 0.001$, ****$P < 0.0001$. Each dot corresponds to one mouse.

Appendix Figure S7

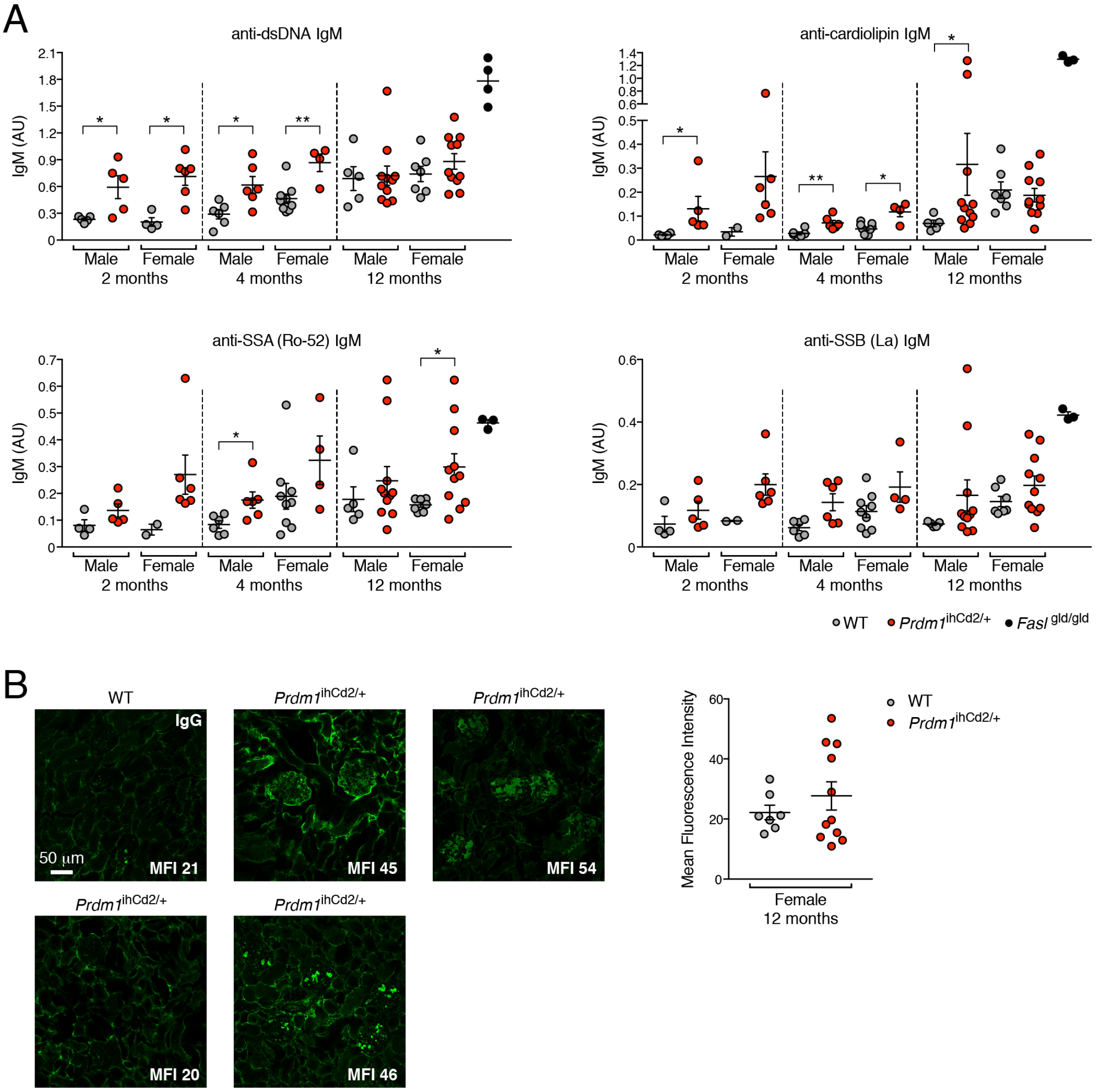

Appendix Figure S7. **Presence of autoantibodies and immune complex deposits in *Prdm1*$^{\text{ihCd2/+}}$ mice.**

(**A**) Presence of IgM antibodies detecting dsDNA, cardiolipin, SSA (Ro-52) and SSB (La) in the serum of *Prdm1*$^{\text{ihCd2/+}}$ (red dots) and wild-type (gray dots) mice at the age of 2, 4 and 12 months. The titers of the different IgM antibodies were determined in the serum of male and female mice by ELISA and are displayed as arbitrary units (AU). The serum of 6-month-old *Fasl*$^{\text{gld/gld}}$ mice was used as positive control. (**B**) Presence of IgG immune complex deposits in the glomeruli of kidneys from *Prdm1*$^{\text{ihCd2/+}}$ female mice. Kidney sections of *Prdm1*$^{\text{ihCd2/+}}$ and wild-type (WT) mice at the age of 12 months were stained with an anti-IgG antibody and analyzed by confocal microscopy (left). The mean fluorescence intensity (MFI) per glomerulus was determined by analyzing at least 12 individual glomeruli of each kidney (left), and the MFI values are indicated for *Prdm1*$^{\text{ihCd2/+}}$ and wild-type mice in the dot plot shown to the right. Statistical data (**A**,**B**) are shown as mean value with SEM and were analyzed by the Mann-Whitney test (**A**) or the Student's *t*-test (**B**); $*P < 0.05$, $**P < 0.01$. Each dot corresponds to one mouse.

Appendix Figure S8

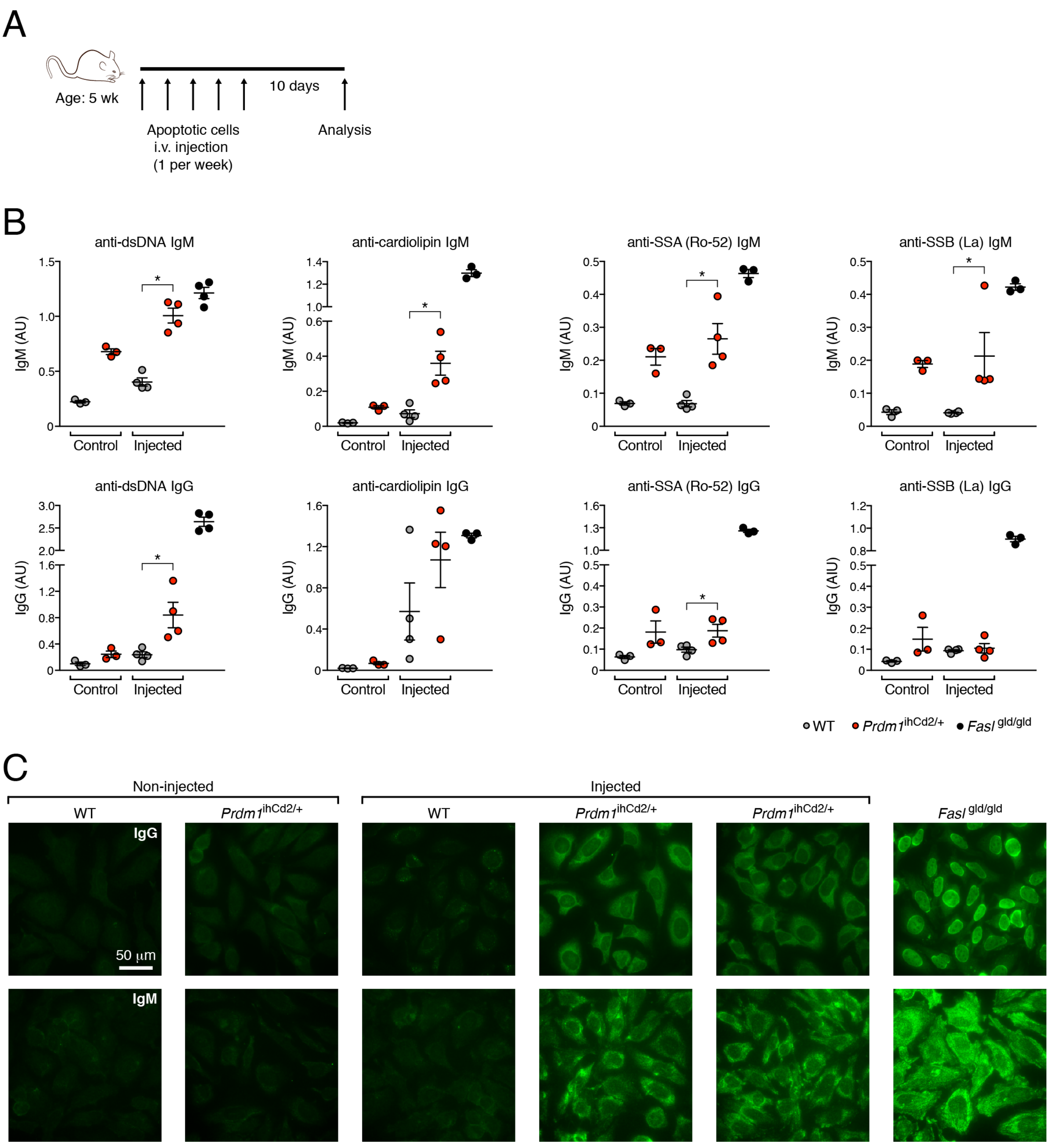

Appendix Figure S8. Accelerated development of autoimmunity in *Prdm1*$^{ihCd2/+}$ mice upon repeated injections of apoptotic cells.

(A) Schematic design of the apoptotic cell injection experiment. Dexamethasone-treated syngeneic thymocytes, which consisted of ~70% apoptotic cells as shown by flow cytometry, were injected intravenously (i.v.) five times at weekly intervals into 5-week-old *Prdm1*$^{ihCd2/+}$ and wild-type mice, as previously published (Duhlin et al., 2016). The injected mice were analyzed 10 days

after the last injection. **(B)** Presence of IgM and IgG antibodies detecting dsDNA, cardiolipin, SSA (Ro-52) and SSB (La) in the serum of injected and non-injected mice at the age of 10 weeks. The titers of the different IgM and IgG antibodies in the serum of *Prdm1*$^{ihCd2/+}$ (red dots) and wild-type (gray dots) mice were determined by ELISA and are displayed as arbitrary units (AU). The serum of 6-month-old *Fasl*$^{gld/gld}$ mice was used as positive control. Statistical data are shown as mean value with SEM and were analyzed by the Mann-Whitney test; $*P < 0.05$. Each dot corresponds to one mouse. **(C)** Detection of anti-nuclear antibodies (ANA) of the IgM and IgG isotype in the serum of injected and non-injected *Prdm1*$^{ihCd2/+}$ or wild-type mice. ANA staining was performed with the serum of the indicated mice by indirect immunofluorescence assay on HEp-2 cells with an Alexa488-conjugated anti-mouse IgM or IgG antibody, respectively. The serum of *Fasl*$^{gld/gld}$ mice was used as positive control.

3. Appendix Supplementary References

Alperovich, G., I. Rama, N. Lloberas, M. Franquesa, R. Poveda, M. Gomà, I. Herrero-Fresneda, J.M. Cruzado, N. Bolaños, M. Carrera, J.M. Grinyó, and J. Torras. 2007. New immunosuppresor strategies in the treatment of murine lupus nephritis. *Lupus* 16:18-24.

Aszódi, A. 2012. MULTOVL: fast multiple overlaps of genomic regions. *Bioinformatics* 28:3318-3319.

Bouvier, G., F. Watrin, M. Naspetti, C. Verthuy, P. Naquet, and P. Ferrier. 1996. Deletion of the mouse T-cell receptor β gene enhancer blocks αβ T-cell development. *Proc. Natl. Acad. Sci. USA* 93:7877-7881.

Chowdhary, V.R., C. Dai, A.Y. Tilahun, J.A. Hanson, M.K. Smart, J.P. Grande, G. Rajagopalan, S.M. Fu, and C.S. David. 2015. A central role for HLA-DR3 in anti-Smith antibody responses and glomerulonephritis in a transgenic mouse model of spontaneous lupus. *J. Immunol.* 195:4660-4667.

Core, L.J., J.J. Waterfall, and J.T. Lis. 2008. Nascent RNA sequencing reveals widespread pausing and divergent initiation at human promoters. *Science* 322:1845-1848.

Driegen, S., R. Ferreira, A. van Zon, J. Strouboulis, M. Jaegle, F. Grosveld, S. Philipsen, and D. Meijer. 2005. A generic tool for biotinylation of tagged proteins in transgenic mice. *Transgenic Res.* 14:477-482.

Duhlin, A., Y. Chen, F. Wermeling, S.K. Sedimbi, E. Lindh, R. Shinde, M.J. Halaby, Y. Kaiser, O. Winqvist, T.L. McGaha, and M.C. Karlsson. 2016. Selective memory to apoptotic cell-derived self-antigens with implications for systemic lupus erythematosus development. *J. Immunol.* 197:2618-2626.

Fuxa, M., and M. Busslinger. 2007. Reporter gene insertions reveal a strictly B lymphoid-specific expression pattern of *Pax5* in support of its B cell identity function. *J. Immunol.* 178:8222-8228.

Gateva, V., J.K. Sandling, G. Hom, K.E. Taylor, S.A. Chung, X. Sun, W. Ortmann, R. Kosoy, R.C. Ferreira, G. Nordmark, I. Gunnarsson, E. Svenungsson, L. Padyukov, G. Sturfelt, A. Jonsen, A.A. Bengtsson, S. Rantapaa-Dahlqvist, E.C. Baechler, E.E. Brown, G.S. Alarcon *et al*. 2009. A large-scale replication study identifies *TNIP1*, *PRDM1*, *JAZF1*, *UHRF1BP1* and *IL10* as risk loci for systemic lupus erythematosus. *Nat. Genet.* 41:1228-1233.

Gu, H., Y.R. Zou, and K. Rajewsky. 1993. Independent control of immunoglobulin switch recombination at individual switch regions evidenced through Cre-*loxP*-mediated gene targeting. *Cell* 73:1155-1164.

Kallies, A., J. Hasbold, D.M. Tarlinton, W. Dietrich, L.M. Corcoran, P.D. Hodgkin, and S.L. Nutt. 2004. Plasma cell ontogeny defined by quantitative changes in Blimp-1 expression. *J. Exp. Med.* 200:967-977.

Langmead, B., C. Trapnell, M. Pop, and S.L. Salzberg. 2009. Ultrafast and memory-efficient alignment of short DNA sequences to the human genome. *Genome Biol.* 10:R25.

Liao, Y., G.K. Smyth, and W. Shi. 2014. featureCounts: an efficient general purpose program for assigning sequence reads to genomic features. *Bioinformatics* 30:923-930.

Love, M.I., W. Huber, and S. Anders. 2014. Moderated estimation of fold change and dispersion for RNA-seq data with DESeq2. *Genome Biol.* 15:550.

Machanick, P., and T.L. Bailey. 2011. MEME-ChIP: motif analysis of large DNA datasets. *Bioinformatics* 27:1696-1697.

Minnich, M., H. Tagoh, P. Bönelt, E. Axelsson, M. Fischer, B. Cebolla, A. Tarakhovsky, S.L. Nutt, M. Jaritz, and M. Busslinger. 2016. Multifunctional role of the transcription factor Blimp-1 in coordinating plasma cell differentiation. *Nat. Immunol.* 17:331-343.

Nutt, S.L., P. Urbánek, A. Rolink, and M. Busslinger. 1997. Essential functions of Pax5 (BSAP) in pro-B cell development: difference between fetal and adult B lymphopoiesis and reduced *V*-to-*DJ* recombination at the *IgH* locus. *Genes Dev.* 11:476-491.

Ogilvy, S., D. Metcalf, C.G. Print, M.L. Bath, A.W. Harris, and J.M. Adams. 1999. Constitutive Bcl-2 expression throughout the hematopoietic compartment affects multiple lineages and enhances progenitor cell survival. *Proc. Natl. Acad. Sci. USA* 96:14943-14948.

Parkhomchuk, D., T. Borodina, V. Amstislavskiy, M. Banaru, L. Hallen, S. Krobitsch, H. Lehrach, and A. Soldatov. 2009. Transcriptome analysis by strand-specific sequencing of complementary DNA. *Nucleic Acids Res.* 37:e123.

Raychaudhuri, S., B.P. Thomson, E.F. Remmers, S. Eyre, A. Hinks, C. Guiducci, J.J. Catanese, G. Xie, E.A. Stahl, R. Chen, L. Alfredsson, C.I. Amos, K.G. Ardlie, BIRAC Consortium, A. Barton, J. Bowes, N.P. Burtt, M. Chang, J. Coblyn, K.H. Costenbader *et al.* 2009. Genetic variants at *CD28*, *PRDM1* and *CD2*/*CD58* are associated with rheumatoid arthritis risk. *Nat. Genet.* 41:1313-1318.

Reimão-Pinto, M.M., V. Ignatova, T.R. Burkard, J.H. Hung, R.A. Manzenreither, I. Sowemimo, V.A. Herzog, B. Reichholf, S. Fariña-Lopez, and S.L. Ameres. 2015. Uridylation of RNA hairpins by tailor confines the emergence of microRNAs in *Drosophila*. *Mol. Cell* 59:203-216.

Revilla-i-Domingo, R., I. Bilic, B. Vilagos, H. Tagoh, A. Ebert, I.M. Tamir, L. Smeenk, J. Trupke, A. Sommer, M. Jaritz, and M. Busslinger. 2012. The B-cell identity factor Pax5 regulates distinct transcriptional programmes in early and late B lymphopoiesis. *EMBO J.* 31:3130-3146.

Schebesta, A., S. McManus, G. Salvagiotto, A. Delogu, G.A. Busslinger, and M. Busslinger. 2007. Transcription factor Pax5 activates the chromatin of key genes involved in B cell signaling, adhesion, migration, and immune function. *Immunity* 27:49-63.

Schwickert, T.A., H. Tagoh, S. Gültekin, A. Dakic, E. Axelsson, M. Minnich, A. Ebert, B. Werner, M. Roth, L. Cimmino, R.A. Dickins, J. Zuber, M. Jaritz, and M. Busslinger. 2014. Stage-specific control of early B cell development by the transcription factor Ikaros. *Nat. Immunol.* 15:283-293.

Shinkai, Y., G. Rathbun, K.-P. Lam, E.M. Oltz, V. Stewart, M. Mendelsohn, J. Charron, M. Datta, F. Young, A.M. Stall, and F.W. Alt. 1992. RAG-2-deficient mice lack mature lymphocytes owing to inability to initiate V(D)J rearrangement. *Cell* 68:855-867.

Smith, K.G., A. Light, G.J. Nossal, and D.M. Tarlinton. 1997. The extent of affinity maturation differs between the memory and antibody-forming cell compartments in the primary immune response. *EMBO J.* 16:2996-3006.

Takahashi, T., M. Tanaka, C.I. Brannan, N.A. Jenkins, N.G. Copeland, T. Suda, and S. Nagata. 1994. Generalized lymphoproliferative disease in mice, caused by a point mutation in the Fas ligand. *Cell* 76:969-976.

Trapnell, C., L. Pachter, and S.L. Salzberg. 2009. TopHat: discovering splice junctions with RNA-Seq. *Bioinformatics* 25:1105-1111.

Wagner, G.P., K. Kin, and V.J. Lynch. 2012. Measurement of mRNA abundance using RNA-seq data: RPKM measure is inconsistent among samples. *Theory Biosci.* 131:281-285.

Weening, J.J., V.D. D'Agati, M.M. Schwartz, S.V. Seshan, C.E. Alpers, G.B. Appel, J.E. Balow, J.A. Bruijn, T. Cook, F. Ferrario, A.B. Fogo, E.M. Ginzler, L. Hebert, G. Hill, P. Hill, J.C. Jennette, N.C. Kong, P. Lesavre, M. Lockshin, L.M. Looi, H. Makino, L.A. Moura, and M. Nagata. 2004. The classification of glomerulonephritis in systemic lupus erythematosus revisited. *J. Am. Soc. Nephrol.* 15:241-250.

Wermeling, F., S.M. Lind, E.D. Jordö, S.L. Cardell, and M.C. Karlsson. 2010. Invariant NKT cells limit activation of autoreactive CD1d-positive B cells. *J. Exp. Med.* 207:943-952.

Wöhner, M., H. Tagoh, I. Bilic, M. Jaritz, D. Kostanova Poliakova, M. Fischer, and M. Busslinger. 2016. Molecular functions of the transcription factors E2A and E2-2 in controlling germinal center B cell and plasma cell development. *J. Exp. Med.* 213:1201-1221.

Yang, H., H. Wang, C.S. Shivalila, A.W. Cheng, L. Shi, and R. Jaenisch. 2013. One-step generation of mice carrying reporter and conditional alleles by CRISPR/Cas-mediated genome engineering. *Cell* 154:1370-1379.

Yates, A., W. Akanni, M.R. Amode, D. Barrell, K. Billis, D. Carvalho-Silva, C. Cummins, P. Clapham, S. Fitzgerald, L. Gil, C.G. Giron, L. Gordon, T. Hourlier, S.E. Hunt, S.H. Janacek, N. Johnson, T. Juettemann, S. Keenan, I. Lavidas, F.J. Martin *et al.* 2016. Ensembl 2016. *Nucleic Acids Res.* 44:D710-D716.

Zhang, Y., T. Liu, C.A. Meyer, J. Eeckhoute, D.S. Johnson, B.E. Bernstein, C. Nussbaum, R.M. Myers, M. Brown, W. Li, and X.S. Liu. 2008. Model-based analysis of ChIP-Seq (MACS). *Genome Biol.* 9:R137.

Zhou, X.-J., X.-L. Lu, J.-C. Lv, H.-Z. Yang, L.-X. Qin, M.-H. Zhao, Y. Su, Z.-G. Li, and H. Zhang. 2011. Genetic association of *PRDM1-ATG5* intergenic region and autophagy with systemic lupus erythematosus in a Chinese population. *Ann. Rheum. Dis.* 70:1330-1337.
